# Thyroid Hormone Levels in Obese Children and Adolescents with Non-Alcoholic Fatty Liver Disease

**DOI:** 10.4274/Jcrpe.1155

**Published:** 2014-03-05

**Authors:** Emel Torun, İlker Tolga Özgen, Selim Gökçe, Sinem Aydın, Yaşar Cesur

**Affiliations:** 1 Bezmialem Vakıf University Medical Faculty, Department of Pediatrics, İstanbul, Turkey; 2 Bezmialem Vakıf University Medical Faculty, Department of Pediatric Endocrinology and Metabolism, İstanbul, Turkey; 3 Bezmialem Vakıf University Medical Faculty, Department of Pediatric Gastroenterology, İstanbul, Turkey; 4 Bezmialem Vakıf University Medical Faculty, Department of Radiology, İstanbul, Turkey

**Keywords:** Hepatic steatosis, insulin resistance, thyroid hormones

## Abstract

**Ob­jec­ti­ve**: We aimed to determine the association of thyroid functions with the components of metabolic syndrome (MS) and non-alcoholic fatty liver disease (NAFLD) in pediatric obese patients.

**Methods**: The study included 109 obese children (aged 9-15 years) and a control group of 44 healthy age and gender-matched children of normal weight. NAFLD was diagnosed by conventional ultrasound examination. We assessed the anthropometric data and serum biochemical parameters including lipid profile, alanine aminotransferase (ALT), fasting glucose and insulin levels and thyroid stimulating hormone (TSH), free thyroxine (fT4) and free triiodothyronine (fT3) levels. The homeostasis model assessment of insulin resistance (HOMA-IR) was calculated as a measure of IR.

**Results**: The mean age and gender distributions in the groups were similar (p=0.23). The mean body mass index (BMI) z-scores of obese children with grade 2-3 NAFLD were significantly higher than those of the obese children without hepatic steatosis (p<0.001). Mean ALT, triglyceride (TG) and LDL cholesterol increased and HDL-cholesterol significantly decreased as the hepatic steatosis increased (p<0.05). HOMA-IR levels in obese subjects with grade 2-3 NAFLD were significantly higher than those in both obese children without NAFLD and grade 1 NADFL (p=0.05 and 0.001, respectively). In the obese subjects, TSH levels were increased significantly as the degree of steatosis increased (p=0.04) but fT3 and fT4 levels were not different. In correlation analysis, TSH was significantly correlated with ALT, BMI SDS and the degree of steatosis.

**Conclusions**: Obese children demonstrate an increase in TSH levels as the degree of steatosis increased.

## INTRODUCTION

Obesity is the result of higher dietary energy intake that is higher than the energy requirement. It is a multifactorial medical problem which includes environmental and genetic components (1,2). It is also known that obesity in childhood is related with a high risk of possible metabolic disorders in adulthood (3,4). Non-alcoholic fatty liver disease (NAFLD) is one of these metabolic disorders emphasized in this context because of the possibility of its progressing to chronic liver disease (5,6). NAFLD affects 2.6% to 9.8% of children and adolescents and this ratio increases up to 38% to 77% among obese adults (6,7). NAFLD is a potentially serious and multifactorial condition and is associated with other metabolic disorders such as insulin resistance (IR), hypertension and dyslipidemia (7). Hypothyroidism has also been identified as a factor for the development of NAFLD because of its important role in lipid metabolism (8).

Thyroid hormone levels in childhood obesity reveal a variety of inconsistencies from normal to elevated thyroid stimulating hormone (TSH) levels and normal or elevated free triiodothyronine (fT3) or free thyroxine (fT4) levels (9). In adults, subclinical hypothyroidism was found to be related to NAFLD in a dose-dependent manner (10). The relationships between thyroid hormone levels and NAFLD, IR and other metabolic disorders related to obesity have not been fully explained in children. In this present study, we aimed to investigate the relationship between NAFLD and thyroid functions in obese children and compare the thyroid functions of obese children with NAFLD with obese and non-obese children without NAFLD.

## METHODS

This study included 109 consecutive obese children and a control group of 44 healthy non-obese children. The age of the children ranged from 9 to 15 years. The study participants were recruited from Bezmialem Vakıf University Pediatric Endocrinology and Metabolism Outpatient Clinic between January 2011 and February 2013.

Each participant underwent a detailed physical examination including anthropometric measurements, estimation of degree of obesity, systolic and diastolic blood pressure (SBP and DBP) measurements. Children with syndromic obesity (Prader Willi, Laurence-Moon Biedle syndrome, etc.) were excluded, as were those whose obesity had an endocrine cause such as Cushing’s syndrome or hypothyroidism. Those with systemic conditions, including cystic fibrosis and inflammatory bowel disease, hepatitis, drug use, history of parenteral nutrition, cigarette use, alcohol use and family history of hereditary hyperlipidemia and/or premature atherosclerosis were also excluded. In patients with fatty liver, antibodies against hepatotropic viruses, serum ceruloplasmin and α1 antitrypsin levels, autoantibodies against nuclear smooth muscle and liver-kidney microsomal type-1 antigens were screened to eliminate infectious, metabolic and autoimmune liver pathologies in patients with fatty liver. Estimation of obesity is based on a body mass index (BMI) equal to or greater than the 95^th^ percentile for gender and age, BMI% and BMI standard deviation score (SDS) (11). Standing height was measured to the nearest 0.1 cm with a Harpenden fixed stadiometer. Body weight (kg) was measured on a SECA balance scale to the nearest 0.1 kg, with each subject dressed in light underwear. BMI was calculated by dividing weight by height in meters squired (kg/m^2^). BP was measured three separate times after the children had been sitting for 10 min and the second and third measurements were averaged. Children with SBP and/or DBP greater than 95^th^ percentile -adjusted for height, age and sex- were considered to have high BP (12).

All blood analyses were performed on fasting samples in both the study and control groups. Cholesterol, high-density lipoprotein (HDL), low density lipoprotein (LDL) and triglycerides (TG) were measured using the homogeneous colorimetric enzyme technique (Roche, Cobas 8000, USA). Serum 25-hydroxyvitamin D [25(OH)D] levels were determined using the electrochemiluminescence enzyme immuonassay method (ECLIA, ADVIA Centaur, DPC Co., USA). Glucose was measured by glucose oxidase technique (Siemens Advia 1800, USA) and insulin levels were analyzed by direct chemiluminescence technique (Siemens Centaur, USA). IR was estimated from fasting plasma measurements using homeostasis model assessment of IR (HOMA-IR) [insulin (mU/L) x glucose (mmol/L)/22.5] (13). IR criteria were HOMA-IR >2.5 for prepubertal children and HOMA-IR >4.0 for adolescents (14).

In accordance with the criteria proposed by WHO, TG levels lower than 150 mg/dL, LDL-cholesterol levels lower than 130 mg/dL and HDL-cholesterol levels higher than 40 mg/dL were considered to be normal (11).

Conventional hepatic US was performed by a radiologist using a GE Logic 9 (USA) with convex transducers (frequency bandwidth 3.5 MHz). The radiologist was blinded to the clinical and laboratory data and to the risk factors of the subjects. Before US examination, the participants rested quietly in a temperature-controlled dark room for 10-15 minutes. Ultrasonographic steatosis scores were defined as follows: no steatosis (grade 0): normal liver echotexture; mild steatosis (grade 1): slight, diffuse increase in fine parenchymal echoes with normal visualization of the diaphragm and portal vein borders; moderate steatosis (grade 2): moderate, diffuse increase in fine parenchymal echoes with slightly impaired visualization of the diaphragm and portal vein borders; severe steatosis (grade 3): fine echoes with poor or no visualization of the diaphragm and portal vein borders, or posterior portion of the right lobe (15). The children were classified into four groups according to their ultrasonographic steatosis scores as follows: non-obese controls with no steatosis; obese, non-NAFLD; obese, degree 1 NAFLD and the obese, degree 2-3 NAFLD group.

Diagnosis of metabolic syndrome (MS) was based on a modification of the National Cholesterol Education Program’s Adult Treatment Panel Criteria (16). Because the body proportions normally change during pubertal development and can vary according to the individual variation and since waist circumference is difficult to interpret in children, BMI was used as an index of obesity according to the previously defined criteria (17). MS was defined as the presence of any three of the following five constituent risks: hypertension (elevated BP as SBP or DBP equal or higher than 95^th^ percentile for age and gender); low HDL cholesterol values (below the 5^th^ percentile for age and gender); hypertriglyceridemia (TG above the 95^th^ percentile for age and gender); obesity (BMI equal or higher than 95^th^ percentile for age and gender); and glucose impairment using pediatric reference standards.

Thyroid hormone levels (fT3, fT4 and TSH) were measured using a direct chemiluminescence technique (ADVIA Centaur XP, USA). The norm values for the respective ranges were between 0.8 -5.4 uIU/ml for TSH, between 4.3-8 pmol/L for fT3 and 10.3-25.7 pmol/L for fT4 (18).

Statistical analysis was performed using PASW Statistics, v.13.0. Median and interquartile ranges were used for the metric variables. Mann-Whitney U was used to calculate the difference between two parameters in the groups; the Kruskall Wallis test was used in the calculation of the difference between two parameters in groups with more than two in the same group and between different groups. The Spearman correlation was used for correlation analysis. Categorical data were evaluated using the chi-square test; p<0.05 was accepted as being statistically significant. 

The study was approved by the local ethics committee. Written informed consent was obtained from the parents.

## RESULTS

The study included 24 obese children without NAFLD (11 female), 44 obese children with grade 1 NAFLD (20 female), 38 obese children with grade 2-3 NAFLD (21 female) and 44 non-obese healthy control subjects without NAFLD (20 female). The median age and gender distribution in the groups were similar (p=0.168 and 0.705, respectively). The median BMI SDS value in obese children was significantly higher than that in the control group (p<0.001). The median BMI SDS value was found to be significantly higher in the obese children with grade 2-3 NAFLD compared with those of both the other obese and non-obese subjects (p<0.001) (Table 1).

The HOMA-IR values of children with grade 2-3 NAFLD were significantly higher than those of the control group (p<0.001), the obese group without steatosis and the obese group with grade 1 hepatic steatosis (p<0.05). Median HOMA-IR significantly increased as the degree of steatosis increased. Median ALT levels in the obese subjects with grade 2-3 NAFLD were significantly higher than those in the controls and in the obese children without NAFLD or grade 1 NAFLD (p<0.01). Median total cholesterol levels were similar in all groups (p=0.263). LDL cholesterol and TG levels increased and HDL-cholesterol levels decreased significantly as the grade of the steatosis increased (p<0.001, <0.05 and / <0.05, respectively) (Table 2).

TSH levels in the obese children with grade 2-3 steatosis were found to be significantly higher than those in the control group, the obese-without steatosis group and the obese grade 1 steatosis group (p=0.04). Mean fT4 and fT3 levels did not differ significantly between the groups (p=0.38 and 0.22, respectively) (Table 3).

Median SBP in group 3 was significantly higher compared with the control group, the obese group without steatosis and the obese group with grade 1 steatosis (p<0.001). Median DBP measurements also increased significantly as steatosis increased (p<0.001). MS was noted in 3 of the 23 children (12.5%) in the obese group without steatosis, in 16 of the 43 children (36.2%) in the obese group with grade 1 steatosis and in 13 of the 40 children (34.2%) in the obese group with grade 2-3 steatosis. The occurrence of MS increased as the grade of steatosis increased but did not reach a statistically significant level (p=0.098) (Table 3).

The Spearman correlation of TSH levels with other anthropometric and biochemical parameters showed significant correlation with ALT (rs=0.250, p=0.024), BMI SDS (rs=-0.303, p=0.003) and the degree of steatosis (rs=0.342, p=0.001) (Table 4).

## DISCUSSION

The prevalence of obesity in both children and adults is increasing worldwide, as is the prevalence of its complications related to the endocrinological, cardiovascular and gastrointestinal systems. NAFLD is a clinico-histopathological entity associated with obesity and is a potentially serious condition because of the associated liver-related morbidity and its possibility of progressing to cirrhosis (7). The relationship between obesity, obesity- related NAFLD and thyroid functions in childhood is an issue which should be brought to attention.

Thyroid functions in obesity reveal a variety of inconsistencies, from normal fT3 and fT4 levels to elevated TSH in 10-23% of obese subjects (19). This entity defined as subclinical hypothyroidism is thought to be related to the resistance to thyroid hormones of peripheral tissues and a decreased negative feedback relationship between TSH and peripheral thyroid hormones (20). Subclinical hypothyroidism is also reported to be related to the relative low level of T4 binding protein in obese children (21). The relationships between NAFLD and IR, hypertension and hyperlipidemia have been fully determined but limited studies have been conducted on the effect of hepatic steatosis on thyroid functions in children.

In adults, the first reports pointing to the association between thyroid functions and liver enzyme activity were conducted by Targher et al (22) and emphasized by Itterman (23). One of the most comprehensive studies was conducted by Chung et al (10) in adults and this study revealed that the prevalence of US-diagnosed NAFLD and abnormal ALT levels increased steadily with increasing grades of hypothyroidism. In studies including subjects with biopsy-proven nonalcoholic steatohepatitis (biopsy-proven NASH), subjects with hypothyroidism were found to be more likely to have NAFLD than those without hypothyroidism, even after eliminating the impact of other metabolic disorders combined with obesity such as diabetes, hypertension, hyperlipidemia (24). In another study, Carulli et al (25) found that euthyroid patients with biopsy-proven NASH had higher TSH levels compared with those with simple steatosis. In the study conducted by Pacifico et al (26) consisting of 402 overweight and obese children with NAFLD, the authors tested the hypothesis that thyroid dysfunction might be associated with an increased risk of NAFLD. The results of this study revealed that elevated TSH concentrations were significantly associated with an increased prevalence of hepatic steatosis and elevated ALT concentrations. High TSH levels were associated with increased odds of having hepatic steatosis, hepatic steatosis with elevated ALT, hypertriglyceridemia, elevated total cholesterol and IR after adjustment for age, gender, pubertal status and BMI SDS (26). In our study we investigated the relationship of all the components of MS with degree of hepatic steatosis as well as thyroid functions. In our study, the median LDL-cholesterol, TG, IR and ALT levels were increased and HDL-cholesterol decreased as the hepatic steatosis degree increased. TSH levels -but not fT3 or fT4- were also elevated with increase in the degree of obesity and steatosis. The increase in the TSH levels was correlated with the grade of obesity, grade of steatosis, ALT levels. Within these parameters, the grade of steatosis displayed the strongest correlation. These findings show that the effect of steatosis on TSH levels is not independent from other factors such as obesity and serum lipid levels. Besides, some other factors that were not evaluated in the present study may have an effect on TSH levels.

The relationship between TSH levels and serum lipid profile in obese children has also been investigated. Results of these studies revealed that patients with hypothyroidism have hyperlipidemia, decreased fatty acid oxidation and decreased hepatic output of TG (27,28). In addition, hypothyroidism was found to be associated with lipid peroxidation, one of the leading candidates for cellular injury in patients with hepatosteatosis (29). In our study, the grade 2-3 NAFLD obese group had significantly higher levels of LDL-cholesterol and TG and lower HDL cholesterol levels compared with the obese- non NAFLD and the lean group. These findings showed that the severity of steatosis was correlated with most of the metabolic dysfunctions such as hyperlipidemia in the severely obese group and with higher TSH levels as well. Studies on the relationship between serum lipid profile and TSH levels have revealed that HDL cholesterol was lower in NAFLD obese children with hypothyroidism compared with NAFLD obese children without hypothyroidism (30). In our study, HDL cholesterol was lower in the grade 2-3 NAFLD obese group compared with the obese non-NAFLD and the lean groups, but the differences did not reach a significant level.

SBP and DBP values were found to be higher in the obese NAFLD group as compared with the non-NAFLD group and the lean group. Increased risk of peripheral vascular resistance, increased arterial stiffness and endothelial dysfunction was reported in obese NAFLD children in previous studies (31). Hypertension has also been reported in some studies with subclinical hypothyroidism (29). In our study, both SBP and DBP increased with increase in degree of obesity and increase in severity of steatosis but no correlation was found between BP and TSH levels. 

IR, leading to impaired hepatic glucose production and glucose uptake in muscle is a component of the MS. Recent studies have revealed that subclinical hypothyroidism worsens IR (32). It has been reported that increasing levels of TSH and decreasing levels of fT4 are associated with increased IR (29). In our study, IR increased as the degree of steatosis and obesity increased parallel to increased TSH levels but the relationship between TSH and IR disappeared in correlation analysis.

One of the limitations of the present study is its cross-sectional design which permitted only an examination of association, not causation. In addition, some of the observed differences in IR between the study groups may be attributed to gender (which though statistically insignificant, was not exactly matched) and to differences in pubertal stage (which was not assessed). The diagnosis of NAFLD was based on ultrasound data and the severity of liver disease was not confirmed histologically.

In conclusion, our study shows that TSH levels increased as the grade of the steatosis increased. Hypothyroidism might not commonly lead to NAFDL in obese patients but appears to contribute to the degree of the obesity, grade of the steatosis and to the development of the major components of MS.

## Figures and Tables

**Table 1:**
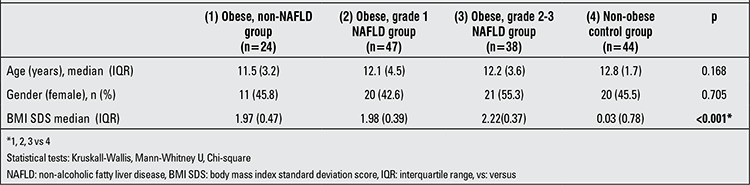
Demographic and anthropometric characteristics of the study and control groups

**Table 2:**
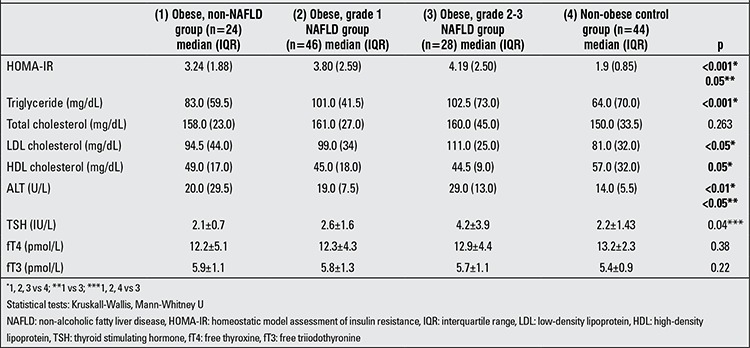
Biochemical characteristics of the study and control group

**Table 3:**
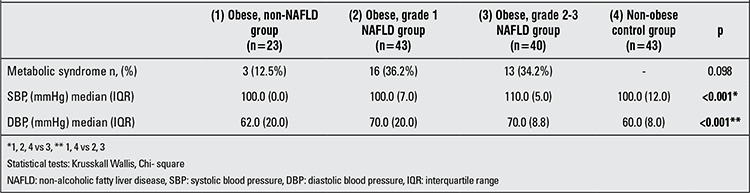
Clinical and laboratory data of the study and control groups

**Table 4:**
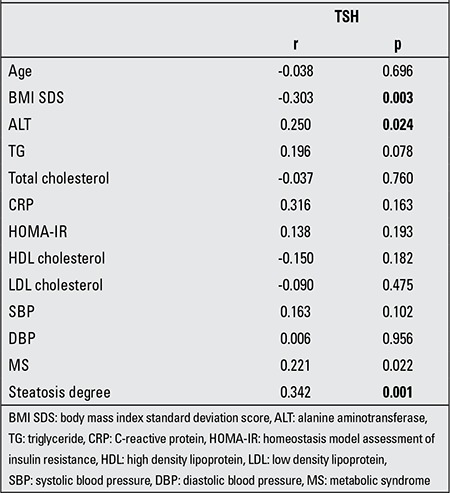
The non-parametric correlation analysis of thyroid stimulating hormone (TSH) levels with anthropometric and biochemical features (Spearman’s correlation)
